# It’s uncomplicated: Prevention of urinary tract infections in an era of increasing antibiotic resistance

**DOI:** 10.1371/journal.ppat.1011930

**Published:** 2024-02-01

**Authors:** Lori L. Burrows

**Affiliations:** Department of Biochemistry and Biomedical Sciences and the Michael G. DeGroote Institute of Infectious Disease Research, McMaster University, Hamilton, Ontario, Canada; Duke University School of Medicine, UNITED STATES

## Infections of the urinary tract are common

Urinary tract infections (UTIs) rank near the top of the long list of medical issues that can affect humans. They are the most common reason for outpatient visits in the United States (US), and clinics around the world see millions of cases annually. UTIs can be broadly categorized as uncomplicated infections that are usually straightforward to resolve, versus complicated UTIs that because of the pathogen, anatomy or health status of the patient, and/or presence of medical devices such as urinary catheters or stents, are more challenging to resolve. Complicated UTIs are associated with formation of antibiotic-tolerant bacterial or fungal biofilm communities on medical devices during prolonged use, and risk is correlated with duration of device placement [1]. Although uncomplicated UTIs are relatively simple to treat, they can be recurrent, defined as 2 or more symptomatic infections in 6 months, or 3 in 12 months, often with the same pathogen [2]. If inadequately treated, both types of UTIs can progress to severe disease, including kidney damage and sepsis, with the urinary tract being a major source of bloodstream infections [3,4].

## Uncomplicated UTIs disproportionately affect women

More than half of adult women will have an uncomplicated UTI at some point in their lives, and risk is correlated with increasing age, with a prevalence of approximately 20% in those over 65 years [2]. Between 20% and 30% of women will experience a recurrence of their initial UTI within 3 to 4 months [5]. These numbers are likely an underestimate, because UTIs are not reportable in most jurisdictions. Women are more susceptible than men due to specific anatomical and physiological differences [6]. In women, the urethral opening is closer to the anus, a primary source of bacteria that can cause uncomplicated UTIs. The shorter female urethra means bacteria have less distance to travel from the external opening to reach the bladder. Sexual activity can introduce bacteria into the urethra, and women are more likely to get UTIs after intercourse than men [7]. Changes in function of the urogenital tract related to pregnancy, menopause, or use of diaphragms or spermicidal agents for birth control can increase the risk for infection [8]. For both men and women, conditions that prevent the bladder from emptying completely—including prolapsed uterus or bladder, the presence of kidney or bladder stones, or medical devices—can lead to urine retention and increased infection risk. Elderly adults or people with diseases such as diabetes, in whom immune function is decreased, are also more susceptible to UTIs [4].

## UTIs are costly—lost productivity, repeated exposure to antibiotics, and resistance

UTIs are an inconvenience to the individual, but more importantly, have substantial economic, societal, and health repercussions, costing billions of dollars in the US alone [9,10]. From lost productivity due to missed work, to the frequent use of hospital emergency departments and antibiotics, UTIs have broad impact [11]. Repeated antibiotic exposure following recurrent infections can lead to urogenital and gut dysbiosis, and potentially other health issues due to perturbation of the microbiome [12]. More alarmingly, the widespread use of broad-spectrum antibiotics to treat recurrent UTIs has helped make antibiotic resistance in common uropathogens a grim reality [13–15].

## Most uncomplicated UTIs are caused by *E*. *coli*

Though many pathogens can cause UTIs, *Escherichia coli* is the prime suspect, causing approximately 80% of uncomplicated cases [10]. Although some strains of this bacterium are peaceful residents of our gut, others can wreak havoc when they find their way into the urinary tract. Uropathogenic *E*. *coli* express hair-like structures called fimbriae that bristle from their surface. At the tip of each fimbria is a protein called FimH that has high affinity for D-mannose sugars, particularly α-1,3 linked mannosides that are abundantly displayed on urothelial cells lining the urethra and bladder [16]. By latching onto these sugars, *E*. *coli* resists being swept away by the shear forces generated during urination, allowing it to initiate infection. FimH has an unusual structure that causes it to clamp down more tightly (up to 3 orders of magnitude) onto its receptor when moderate forces are applied [17]. This mechanism is called a catch-bond and allows the bacteria to tune their adherence in response to forces in the local environment. FimH can also interact with TLR-4, a host receptor that plays a crucial role in our immune response to bacteria. These interactions can contribute to uncomfortable symptoms because they increase inflammation but the inflammatory response may also help to impair colonization. The bacteria can invade the urothelium in a FimH-dependent manner, leading to the formation of intracellular bacterial colonies that are difficult to clear and potentially responsible for seeding recurrent infections [5].

In the past, *E*. *coli* infections were easily treated with antibiotics, but over the last 3 decades its susceptibility to commonly prescribed drugs has decreased, particularly in parts of the world where antimicrobial resistance is more prevalent [18]. *E*. *coli* was the most frequently identified pathogen in a recent global analysis of deaths attributed to or associated with antimicrobial resistance [15]. In countries with robust stewardship practices, resistance levels have remained relatively stable. A Dutch study compared changes in the levels of antibiotic resistance between 2004 and 2014 in *E*. *coli* isolates from uncomplicated UTIs in women. Susceptibility to common antibiotics was mostly unaffected, but alarmingly, the proportion of strains carrying genes for resistance enzymes of concern (such as extended-spectrum β-lactamases) increased 22-fold during that period [19]. In contrast, Ong and colleagues surveyed more than 700,000 *E*. *coli* UTI isolates over a 5-year period (2014 to 2019) in a European teaching hospital—where more complex presentations might be expected—and saw an increasing trend in antibiotic resistance for commonly used antibiotics [20]. Fortunately, multidrug-resistant strains remain relatively rare, although virulent isolates resistant to 14 of 15 antibiotics have been reported [21], as well as a few *E*. *coli* strains with pan-resistance to carbapenems and last-resort drugs like colistin [22]. Specific molecular signatures have been associated with a subset of multidrug-resistant strains commonly isolated from UTIs, suggesting they have particular advantages in the host environment [23].

## Gird your loins—non-antibiotic strategies to prevent and treat UTIs

As antibiotic resistance continues to escalate, it is unsettling to see that the development of new antibiotics has stalled [24]. A 2022 analysis of guidelines for management of recurrent UTIs reported that continuous or post-intercourse antibiotics continue to be widely recommended [25], a practice that will only contribute to the spread of resistance. This situation highlights the pressing need for non-antibiotic strategies to prevent uncomplicated UTIs. Behavioral changes, dietary interventions, or innovative medical approaches such as sublingual vaccines [26] could keep UTIs at bay without the need to reach for antibiotics. Clinical trials of non-antibiotic interventions for UTIs are underway in multiple countries. **Table 1** summarizes available strategies for prevention and treatment of uncomplicated UTIs; below I focus on vaccination and the role of D-mannose.

**Table 1:** Prevention and treatment strategies for uncomplicated UTIs.

Treatment/approach	How does it work?	References
**PREVENTION**		
D-mannose	Prevents the adhesion of *E*. *coli*, the most common UTI-causing bacteria, to the urothelium	[27]
Mannosides	Chemically modified versions of mannose that have high affinity for the FimH protein on *E*. *coli* fimbriae, blocking adhesion to the urothelium	[28]
DAPAD complex	A mixture of D-mannose, citric acid, prebiotic fiber, *Astragalus*, and dandelion	[29]
Cranberry juice or supplements	Contains D-mannose and proanthocyanidins that may prevent bacteria from binding to the urothelium	[30]
Probiotics	Beneficial bacteria such as *Lactobacilli* restore the vaginal flora and may prevent the growth of pathogenic bacteria causing UTIs	[31]
Vaccines	Vaccines against common UTI pathogens are in development and could prevent recurrent infections	[26]
Topical estrogen	For postmenopausal women, might help rebalance the vaginal flora and reduce the risk of recurrent UTIs	[25]
Herbal remedies Uva Ursi (Bearberry) Goldenseal Horsetail	Antimicrobial and diuretic properties Contains berberine with antimicrobial effects Used as a diuretic to flush out bacteria	[32]
Drinking extra water	Helps flush out bacteria due to more frequent urination	[33]
Hygiene practices	Wiping front to back, and urinating before and after intercourse can reduce UTI risk	[7]
**TREATMENT**		
Antibiotics	Inhibit the growth of bacteria; many uropathogens are becoming resistant to commonly available drugs	[25]
Bacteriophages (phages)	Viruses that infect and kill bacteria. Show promise as an antibiotic alternative, especially for resistant strains, but their host specificity requires careful matching of bacterium to phage	[34]
Methenamine hippurate	An older drug that is converted to formaldehyde in the urine and acts as an antiseptic, killing bacteria	[25]

## The intention is prevention

Vaccination is a time-tested and reliable way to prevent infectious diseases, and there have been numerous attempts, particularly in Europe, to develop anti-UTI vaccines. A 2020 systematic review of clinical trials that investigated approaches for managing recurrent UTIs concluded that vaccines could reduce recurrence and the need for antibiotics [35]. Because UTIs occur at mucosal surfaces, it is important that vaccination strategies are designed in a way that generates appropriately targeted immune responses. To that end, an oral vaccine (MV140/Uromune, Inmunotek S.L., Spain) demonstrated efficacy in preventing recurrent UTIs in recent Phase 2/3 clinical trials across 26 countries [26]. The vaccine contains a heat-killed mixture of 4 different uropathogens, including *E*. *coli*, and is administered under the tongue as a spray, making it easy for patients to take at home. One limitation of this vaccine is the lengthy regimen—daily doses for 3 months—needed to stimulate effective immunity.

Among the simplest interventions for prevention of UTIs is the dietary intake of cranberry or D-mannose, an inexpensive, simple sugar found in many fruits—including cranberries [27]. A recent Cochrane review supported the use of cranberry products for reducing the risk of symptomatic, culture-verified UTIs in women with recurrent UTIs [30]. Unfortunately, not all cranberry-containing products are equally effective because of variations in the ways they are formulated and how much of the active ingredients (proanthocyanidins and D-mannose) they ultimately contain, potentially misleading consumers into using ineffective products.

**Fig 1:**
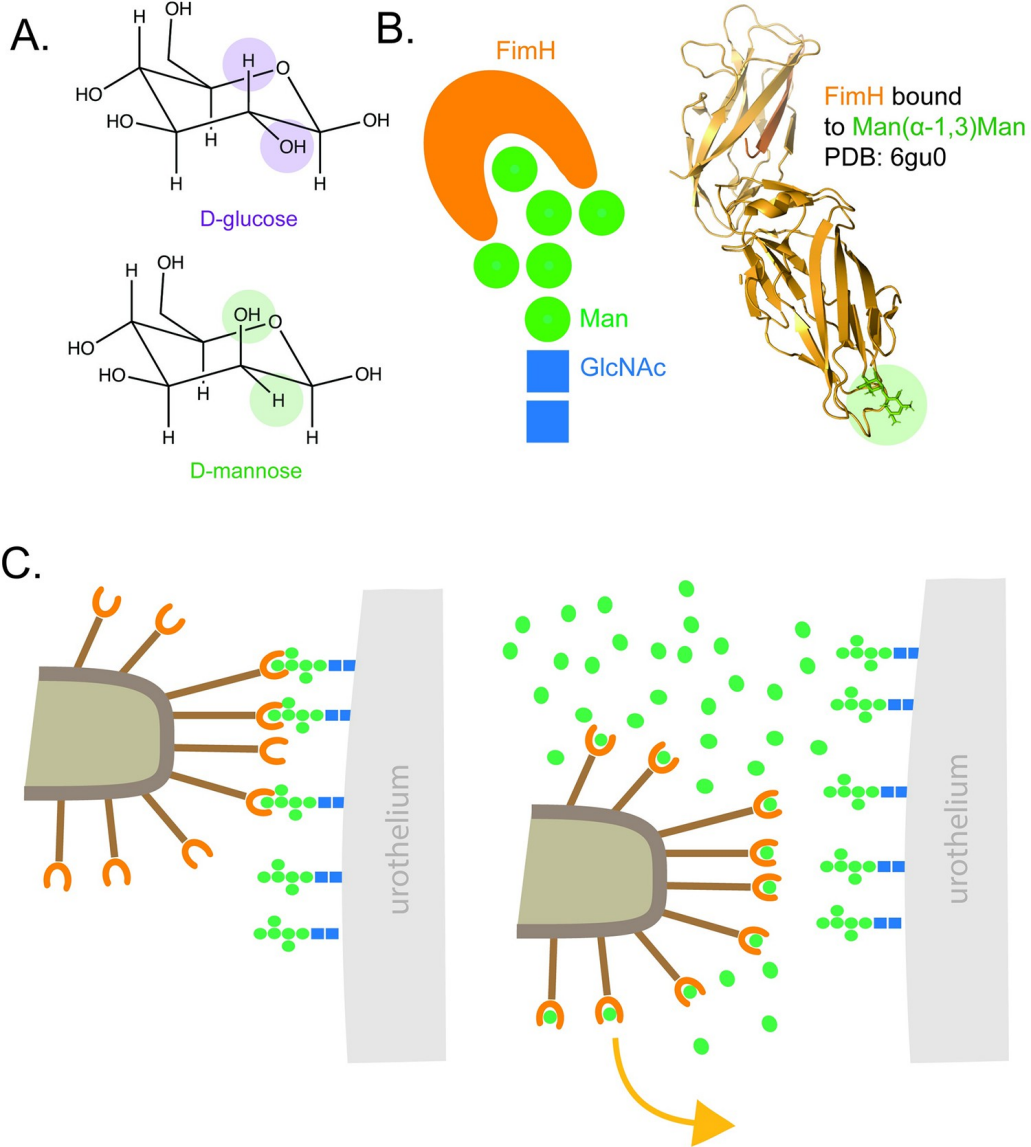
*E*. *coli* binds to D-mannose using FimH. **(A)** D-mannose is a monosaccharide that—unlike its structural relative, glucose—is poorly metabolized by humans and accumulates in urine. Structural differences between D-glucose and D-mannose are highlighted. **(B)** Not-to-scale schematic of FimH interacting with a mannose oligosaccharide similar to those present on human urothelium is shown on the left, and a structural model of the FimH protein bound to the high affinity receptor Man-α-1,3-Man is shown on the right for scale [17]. **(C)** *E*. *coli* uses the FimH adhesin on its type I fimbriae to bind to mannosides on the urothelium to establish infection. Excess free D-mannose (green circles) in the urine from dietary supplementation competes for FimH, preventing interaction of the bacteria with the urothelium and allow its elimination through urination.

A more reliable dietary intervention—because it is available as a highly purified active material—is D-mannose itself (**Fig 1A**). Unlike sugars such as glucose, D-mannose is poorly metabolized by humans, does not raise blood sugar, and ends up in the gut and urine where it is eventually eliminated. This makes it safe for use in people with diabetes, and it is generally well-tolerated with mild gastrointestinal symptoms in some subjects. D-mannose is proposed to prevent UTIs by inhibiting *E*. *coli* adhesion. High levels of free D-mannose in urine compete for the binding pocket of FimH (**Fig 1B**), blocking bacterial adherence to the urothelium and causing the pathogen to be flushed from the bladder by urination (**Fig 1C**). One of the limitations of using D-mannose as a preventative is that its mechanism of action primarily targets *E*. *coli*, making it less effective against UTIs caused by other species. However, unlike the use of antibiotics, this anti-adhesion approach is considered less likely to select for resistance, and thus safer from a public health perspective [36]. Other approaches to blocking adhesion include small molecule “pilicides” that disrupt fimbrial assembly, but none of these have progressed into clinical use [6].

## But does it work? Clinical evidence on use of D-mannose for uncomplicated UTIs

Solid clinical evidence, particularly rigorous, large-scale trials examining the efficacy of D-mannose versus placebo for prevention of UTI, remains limited [37]. However, a number of studies have reported success in reducing the incidence of uncomplicated recurrent UTIs.

Kranjčec and colleagues [38] divided 308 women with recurrent UTIs into 3 treatment arms: D-mannose powder, the antibiotic nitrofurantoin, or no treatment. D-mannose reduced the risk of recurrent UTIs to levels similar to the antibiotic, and both were more effective than receiving no treatment. A randomized crossover pilot study of 60 women with recurrent UTIs found that the mean time to recurrence was almost 4 times longer with D-mannose (200 days) versus antibiotic treatment (53 days) [39]. Another 2016 pilot study [40] explored use of D-mannose in conjunction with antibiotics for the treatment of acute UTIs in women. The researchers reported a potentially synergistic effect, leading to faster symptom resolution and decreased recurrence risk.

Some groups are studying D-mannose in combination with other components to treat, rather than prevent UTIs. For example, Salvatore and colleagues [29] recently reported the results of a single-center, randomized, double-blind, placebo-controlled trial in 70 women (35 per group) of DAPAD, a mixture of D-mannose, citric acid, prebiotic fiber, *Astragalus*, and dandelion, for the oral treatment of uncomplicated UTI. They saw clinical resolution in a third of treated patients versus none in the placebo group at 6 days (*p* < 0.0001) and in over 88% of treated patients versus 20% in the placebo group at day 35 (*p* < 0.0001). This study would have benefitted from a D-mannose-only arm for comparison.

## Mannose/mannoside analogs: A superior approach?

Despite the limited clinical trial data available to support the use of D-mannose to prevent UTIs, there is a long history of using this sugar scaffold as a basis for development of new therapies. One drawback of D-mannose is its low micromolar affinity for FimH, so medicinal chemistry efforts have focused on creating orally available mannoside antagonists with nanomolar or sub-nanomolar affinity and better metabolic stability [28]. A major focus has been development of multivalent mannoside analogs with high binding affinity that more closely mimic the presentation of glycan oligomers on the urothelium [41,42]. Clinical trials are still needed to demonstrate whether these molecules would be better than D-mannose at blocking *E*. *coli* adherence. Even if these compounds show enhanced efficacy or other benefits, it is important to consider their potential liabilities in terms of cost and accessibility should they be categorized as drugs. D-mannose is available direct to consumers as a nutritional supplement and regulated as a GRAS (generally regarded as safe) compound. In contrast, synthetic mannoside derivatives may be subject to increased regulatory scrutiny and complex approval pathways, leading to greater cost and more restricted access to the products. On the other hand, the successful development of patentable mannoside analogs with increased potency compared to D-mannose alone might lead to the creation of a reimbursement pathway, especially if such compounds could demonstrate substantial health and economic benefits. This scenario could make synthetic mannoside analogs affordable and more widely available.

## Who pays for something that isn’t “medicine?”

A conundrum of our modern approach to healthcare is the often disproportionate focus on treatment over prevention. This mindset, especially prevalent in North American models, underscores the need to reevaluate and realign our health priorities. While D-mannose and related products show promise in preventing UTIs, they aren’t considered conventional “medicines” and thus do not always receive the funding or attention that they deserve. It can be hard to attract the necessary investments to carry out sorely needed large randomized clinical trials of this type of dietary intervention for prevention of UTIs, but equally difficult to convince public or private health insurers to cover such non-standard treatments in the absence of robust clinical data. The result is that—for now—the cost of these interventions must be borne by the patient. D-mannose from multiple manufacturers is readily accessible to consumers through health food stores and online retailers, but despite the potential benefits, its price may remain a barrier for some.

## Could rapid diagnostics reduce inappropriate antibiotic use?

One unfortunate consequence of the COVID-19 pandemic was the increased use of antibiotics to prevent secondary bacterial infections, with negative impacts on resistance rates [43]. However, it also led to a burst of innovation in the form of new rapid point-of-care (PoC) diagnostic assays. Development of similar quick and easy tests for uncomplicated UTIs that could differentiate between *E*. *coli* versus other pathogens, or even identify strains resistant to commonly prescribed antibiotics [44], could be useful for antibiotic stewardship. The widely used urine dipstick tests report on inflammatory markers rather than pathogens, and have little value in helping physicians choose the right antibiotics for a particular patient, but they’re cheap and have good negative predictive power. More accurate PoC tests that could direct appropriate therapy could reduce the number of mismatches between “bug and drug.” An ideal test would meet the “REASSURED” version of the World Health Organization’s criteria: **R**eal-time, **E**asy-to-collect, **A**ffordable, **S**ensitive, **S**pecific, **U**ser-friendly, **R**apid and robust, **E**quipment-free, and **D**eliverable to end users [45]. Although limited clinical trials of various PoC tests for diagnosis and management of uncomplicated UTIs continue, a recent systematic review concluded that there remain insufficient data to show clear benefits for UTI diagnosis or management compared to traditional culture-based methods [46]. A fulsome analysis of the economic benefits of rapid and accurate diagnosis of uncomplicated UTIs will be important to support broader uptake of PoC tests.

## Conclusions

UTIs remain a pervasive health challenge, and an ongoing and costly liability for individuals, healthcare systems, and economies. An evolving understanding of appropriate, non-antibiotic prophylactic measures and innovative approaches to diagnosis and therapy will help mitigate these issues. By focusing on prevention of UTIs and minimizing antibiotic overuse, we can fight both these common infections and the looming specter of antimicrobial resistance.
